# Phenolic Composition and Biological Properties of *Cynara cardunculus* L. var. *altilis* Petioles: Influence of the Maturity Stage

**DOI:** 10.3390/antiox10121907

**Published:** 2021-11-28

**Authors:** Filipa Mandim, Spyridon A. Petropoulos, Maria Inês Dias, José Pinela, Marina Kostić, Marina Soković, Celestino Santos-Buelga, Isabel C. F. R. Ferreira, Lillian Barros

**Affiliations:** 1 Centro de Investigação de Montanha (CIMO), Instituto Politécnico de Bragança, Campus de Santa Apolónia, 5300-253 Bragança, Portugal; filipamandim@ipb.pt (F.M.); maria.ines@ipb.pt (M.I.D.); jpinela@ipb.pt (J.P.); iferreira@ipb.pt (I.C.F.R.F.); 2 Grupo de Investigación en Polifenoles (GIP-USAL), Facultad de Farmacia, Campus Miguel de Unamuno s/n, Universidad de Salamanca, 37007 Salamanca, Spain; csb@usal.es; 3 Department of Agriculture, Crop Production and Rural Environment, University of Thessaly, 38446 Volos, Greece; 4 Institute for Biological Research “Siniša Stanković”—National Institute of Republic of Serbia, University of Belgrade, Bulevar Despota Stefana 142, 11000 Belgrade, Serbia; marina.kostic@ibiss.bg.ac.rs (M.K.); mris@ibiss.bg.ac.rs (M.S.)

**Keywords:** cardoon, phenolic composition, antioxidant activity, seasonal changes, anti-inflammatory activity, oxidative hemolysis, antimicrobial properties

## Abstract

Hydroethanolic extracts of cardoon petioles collected at sixteen growth stages (P1–P16) were characterized in terms of their phenolic composition and bioactive potential (antioxidant, cytotoxic, anti-inflammatory, and antimicrobial activities). Fifteen phenolic compounds were tentatively identified (i.e., ten phenolic acids and five flavonoid glycosides); the main compounds were 5-*O*-caffeoylquinic and 1,5-di-*O*-caffeoylquinic acids. Samples collected at early maturity (P1–P4) presented a weak positive correlation between the higher content in polyphenols (P3: 101-mg/g extract) and better inhibition capacity against thiobarbituric acid reactive substance formation (TBARS; P3: IC_50_ = 5.0 µg/mL). Samples at intermediate maturation stages (P9) presented higher cytotoxic and anti-inflammatory potential. Moreover, immature petioles showed greater antihemolytic (OxHLIA; P4: IC_50_ = 65 and 180 µg/mL for Δ*t* of 60 and 120 min, respectively) and antibacterial activity. The antifungal activity varied depending on the maturation stage and the fungi strain. In conclusion, the maturation stage may greatly affect the polyphenols composition and content and the bioactive potential of cardoon petioles.

## 1. Introduction

Plant species, including those of the Asteraceae family, contain a massive variety of compounds with high bioactive potential, being considered as the principal sources of new healing agents [1,2]. In particular, representatives of the Asteraceae family have already been characterized by the presence of specific phenolic acids and flavonoids [3]. Despite the significant contributions that compounds of natural origin have made to the discovery of potent drugs, with enormous structural complexity and diversity, their isolation and identification remain an important and rewarding area of study, as new compounds continue to be identified. Plant species remain an excellent source for the discovery of biomolecules with high pharmacological potential [4,5,6]. Plant secondary metabolites show antioxidant, anti-inflammatory, cytotoxic and hepatotoxic, and antimicrobial activities, and several studies are developing nowadays due to a huge plant biodiversity and their secondary metabolites [7,8].

*Cynara cardunculus* L. is a species that belongs to the Asteraceae family, commonly designated as cardoon, and comprises three botanical varieties: var. *altilis* DC, var. *scolymus* (L.) Fiori, and var. *sylvestris* (Lamk) Fiori. This species is widely used in Mediterranean cuisine and folk medicine due to its nutritional composition, choleretic, hypocholesterolemic, and diuretic properties and effectiveness in the treatment of hepatic diseases [9,10]. Cardoon is also an important source of components such as fiber, carbohydrates, inulin, minerals, and polyphenolic compounds [2,10,11]. Besides its nutritional and phytochemical interest, this species is used in a wide variety of industrial applications. For example, it can be used as vegetable rennet in the production of some protected designation of origin (PDO) cheeses [12,13], paper pulp [14], food oil [15], and bioenergy [16,17], as well as animal forage [18,19]. The multifaceted industrial applications of cardoon are fundamental for its economic valorization and exploitation [17]. However, industrial processing generates a large amount of wasted material, which can be an important source of biologically active compounds [17,20]. Since several parameters influence the chemical composition and bioactive properties of the species (i.e., environmental conditions, harvest time, genetic variability, and plant tissue) [10,21,22], the proper exploration and characterization of the species and all its constituents are extremely important and of great interest.

The harvesting and maturity stages are essential for the chemical composition of natural products obtained from plant tissues, as already reported in the literature for various plant species, e.g., flowers and leaves of *Brassica rapa* [23], heads and bracts of cardoon [22,24], leaves of *Cichorium spinosum* [25], okra fruit [26], etc. Therefore, the present work aims to evaluate the influence of the harvest date on the phenolic compound’s composition and the in vitro biological properties of cardoon petioles collected throughout the growth cycle (e.g., start of vegetative growth to senescence). The main objectives of the study were: (a) to value cardoon petioles through a more complete characterization of their properties and chemical composition, (b) to determine how the chemical composition and bioactive properties diverge throughout the plant’s growth cycle, and (c) to evaluate the potential to stimulate their exploitation and economic valorization, since it is still an underexplored vegetable tissue.

## 2. Materials and Methods

### 2.1. Plant Material

**Figure 1:**
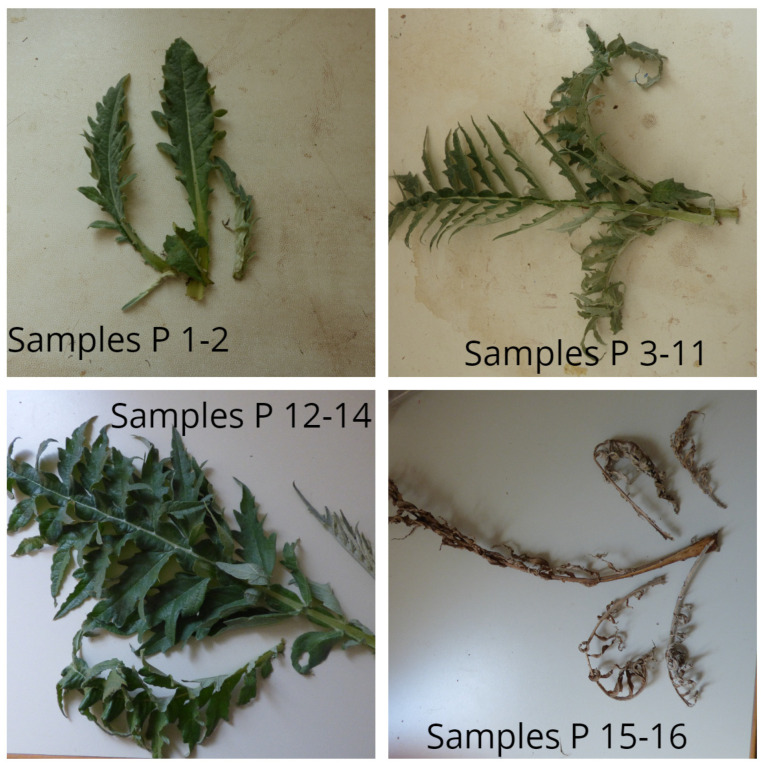
Leaf morphology at different harvesting stages (Sample P 1–16). Photo credits: Petropoulos S.A. (personal record).

Petioles of *Cynara cardunculus* var. *altilis* DC cv. *Bianco Avorio* (Fratelli Ingegnoli Spa, Milano, Italy) were harvested during the growing period of 2017 to 2018 in Central Greece at the experimental field of the University of Thessaly in Velestino (22.756 E, 39.396 N) [22]. Petioles were collected at sixteen harvesting dates according to the principal growth stages (PGS) defined by the Biologische Bundesanstalt, Bundessortenamt, CHemische Industrie (BBCH) scale, comprising the stages between PSG 1 and PSG 9 [27]. Samples P1, P2, and P3 were collected in September, October, and the start of November (all PSG 1), respectively; P4 was collected at the end of November (PSG 2); samples P5, P6, P7, and P8 were collected at the beginning of January (PSG 3), February (PSG 3/4), March (PSG 4), and April (PSG 4/5), respectively; sample P9 was collected at the end of April (PSG 5); samples P10 and P11 were collected at the beginning (PSG 5/6) and at the end of May (PSG 6); P12 was collected at the beginning of June (PSG 6/7); samples P13 and P14 were collected at the beginning (PSG 7/8) and at the end of July (PSG 8); and samples P15 and P16 were collected at the beginning (PSG 8/9) and at the end of August (PSG 9). At each harvesting date, one leaf per plant from 15 individual plants (*n* = 15) was collected based on leaf phenology and according to the principal growth stages defined by Archontoulis et al. [27]. The morphology of leaves at different harvesting stages is presented in Figure 1. For each harvesting date, all the collected leaves were pooled into a batch sample. Each pooled sample consisted of at least 500 g of fresh tissue.

After collection, the leaves were thoroughly cleaned with distilled water, then cut into small pieces and stored in air-sealed plastic bags at deep-freezing conditions. All the samples were freeze-dried (Sublimator model EKS, Christian Zirbus Co., Brunswick, Germany) and reduced with a domestic blender to a fine powder (~20 mesh). The ground samples were stored in air-sealed bags in a deep freezer (−80 °C) and under protection from light until further analysis.

### 2.2. Extraction Procedure

Petiole samples (1.5 g of fine powder) were extracted with a mixture (EtOH/H_2_O, 80:20, *v*/*v*; 30 mL) under dynamic stirring (150 rpm) for 1 h at room temperature and according to the procedure previously described [22]. The obtained solutions were filtered through Whatman No. 4 filter paper and concentrated at 40 °C under reduced pressure (rotary evaporator Büchi R-210, Flawil, Switzerland). The aqueous phase was frozen and lyophilized (FreeZone 4.5, Labconco, Kansas City, MO, USA). The extraction yield of each sample, referring to the concentration of mg of compound/100 g of petioles, was also calculated and expressed in percentage (%).

### 2.3. Phenolic Compound Identification and Quantification

The obtained petiole extracts were redissolved in EtOH:H_2_O (80:20, *v*/*v*) to obtain a final concentration of 10 mg/mL and filtered using 0.22-µm nylon syringe filters. The polyphenolic composition was analyzed by high-performance liquid chromatography coupled to a diode array detector and electrospray ionization mass spectrometry (HPLC-DAD-ESI/MS), according to the chromatographic conditions described [28]. The chromatographic separation was performed using a Waters Spherisorb S3 ODS-2C18 (3 µm, 4.6 mm × 150 mm, Waters, Milford, MA, USA) column at 35 °C and an isocratic elution gradient between 0.1% formic acid in water and 100% acetonitrile. The DAD was programmed at 280 nm, 330 nm, and 370 nm as the preferred wavelengths for the double online detection of protocatechuic acid, hydroxycinnamic acids, and flavonoids, respectively. A Linear Ion Trap LTQ XL mass spectrometer (ThermoFinnigan, San Jose, CA, USA) equipped with an ESI source was used for MS detection in the negative mode, using nitrogen as the sheath gas (50 psi) and operating with a spray voltage of 5 kV, a source temperature of 325 °C, a capillary voltage of −20 V, and a collision energy used of 35 (arbitrary units). The data acquisition was carried out with the Xcalibur^®^data system (ThermoFinnigan, San Jose, CA, USA).

The tentative identification of the phenolic compounds in the studied cardoon petioles was performed by comparison of the chromatographic data (retention times, UV–Vis, and mass spectra) with commercial standards and literature information. The quantification was performed through the determination of the peak areas. Seven-level calibration curves (EtOH:H_2_O, 80:20, *v*/*v*, linearity 80–1.25 µg/mL) prepared for each available commercial standard (Extrasynthèse, Genay, France) were used: compounds number **1**, **3**, **4**, **5**, **7**, **10**, **11**, **12**, and **15**—chlorogenic acid (y = 168,823x + 161,172, *R*^2^ = 0.9999, LOD 0.20 µg/mL, LOQ 0.68 µg/mL); compound number **2**—protocatechuic acid (y = 214,168x + 27,102, *R*^2^ = 0.9999, LOD 0.14 µg/mL, LOQ 0.64 µg/mL); and compounds number **6**, **8**, **9**, **13**, and **14**—quercetin-3-*O*-glucoside (y = 34,843x − 160,173, *R*^2^ = 0.9998, LOD 0.21 µg/mL, LOQ 0.71 µg/mL). The results were expressed as mg equivalents of the corresponding standard used for quantification per g of extract; however, for simplification purposes, the results will be expressed in mg per g of extract.

### 2.4. Evaluation of the Bioactive Potential

#### 2.4.1. Antioxidant Activity

The antioxidant potential of cardoon petioles was evaluated through two cell-based methodologies: the thiobarbituric acid reactive substances (TBARS) formation and the oxidative hemolysis (OxHLIA) inhibition assays. Trolox (Fisher Scientific, Lisbon, Portugal), a synthetic antioxidant, was used as a positive control.

The TBARS assay was performed according to the procedure previously described [10]. The cardoon petiole extracts were redissolved in water to obtain a solution of 5 mg/mL, which was further diluted in order to obtain the concentration range to be tested (0.0012–1.25 mg/mL). The results were expressed as the concentration of extract (IC_50_ µg/mL) responsible for 50% of the oxidation process inhibition.

For the OxHLIA assay, it measured the capacity of the extracts to inhibit oxidative hemolysis using erythrocytes isolated from sheep blood, following the procedure previously described [29]. The results were expressed as the extract concentrations (IC_50_ µg/mL) necessary to ensure 50% of the erythrocyte population integrity after Δ*t* of 60 and 120 min. Phosphate-buffered saline (PBS; pH 7.4) was used as a negative control.

#### 2.4.2. Anti-Inflammatory Activity

The anti-inflammatory activity was determined through the measurement of the petiole extract’s capacity to inhibit the proinflammatory nitric oxide (NO) production in a murine macrophage cell line (RAW 264.7) induced by the lipopolysaccharide (LPS) (Sigma-Aldrich, St. Louis, MO, USA) according to the procedure previously described [29]. The cardoon petiole extracts were redissolved in water to obtain a stock solution with a concentration of 8 mg/mL that was further diluted to obtain the tested concentrations (6.25–400 µg/mL). The commercial anti-inflammatory agent dexamethasone (Sigma-Aldrich, St. Louis, MO, USA) was used as a positive control, and cells without LPS were considered as the negative control. The results were expressed as the extract concentrations (IC_50_ µg/mL) responsible for inhibiting the production of the inflammatory process mediator (NO) by 50%.

#### 2.4.3. Cytotoxic and Hepatotoxic Activities

The cytotoxic and hepatotoxic potential of cardoon petiole extracts were measured using the sulforhodamine B assay according to the procedure previously described [10]. The extracts were redissolved in water, obtaining a stock solution with a concentration of 8 mg/mL that was further diluted to obtain the range of concentrations tested (6.25–400 µg/mL). The cytotoxic activity was measured using four human tumor cell lines: breast carcinoma (MCF-7), non-small cell lung cancer (NCI-H460), cervical carcinoma (HeLa), and hepatocellular carcinoma (HepG2), all acquired from the Leibniz-Institute DSMZ–German Collection of Microorganisms and Cell Cultures GmbH (Braunschweig, Germany). The hepatotoxic activity was evaluated in a nontumor porcine liver primary culture (PLP2). Ellipticine (Sigma-Aldrich, St. Louis, MO, USA) was used as a positive control, and cell suspensions without any sample were used as a negative control. The results were expressed as the extract concentrations (GI_50_ µg/mL) responsible for inhibiting cell proliferation by 50%.

#### 2.4.4. Antimicrobial Activity

Cardoon petiole extracts were redissolved in 5% of dimethyl sulfoxide (DMSO) (Sigma-Aldrich, St. Louis, MO, USA) and diluted to obtain the range of concentrations tested. The antibacterial activity and antifungal potential were evaluated according to the procedure previously described [30]. The antibacterial activity was evaluated against Gram-positive *Bacillus cereus* (food isolate), *Staphylococcus aureus* (ATCC 11632), and *Listeria monocytogenes* (NCTC 7973) and the Gram-negative *Enterobacter cloacae* (human isolate), *Escherichia coli* (ATCC 35210), and *Salmonella* Typhimurium (ATCC 13311). The evaluation of the antifungal potential was assessed against *Aspergillus fumigatus* (human isolate), *Aspergillus versicolor* (ATCC 11730), *Aspergillus niger* (ATCC 6275), *Penicillium funiculosum* (ATCC 36839), *Penicillium ochrochloron* (ATCC 9112), and *Penicillium aurantiogriseum* (*Penicillium verrucosum* var. *cyclopium*; food isolate). All the microorganisms are deposited at Mycological Laboratory, Department of Plant Physiology, Institute for Biological Research “Siniša Stanković”, National Institute of Republic of Serbia, University of Belgrade, Serbia. Commercial antibiotics ampicillin and streptomycin were used as the positive controls with the antifungal ketoconazole (all acquired from Sigma-Aldrich, St. Louis, MO, USA). A solution of 5% DMSO was used as the negative control. The results were presented as the minimal inhibitory (MIC), bactericidal (MBC), or fungicidal (MFC) concentration (mg/mL).

### 2.5. Statistical Analysis

All the assays were performed in triplicate, while three extracts were obtained from each sample. The results were presented as the mean value ± standard deviation (except for the antimicrobial activity), both calculated using Microsoft Excel (Microsoft Corporation, Redmond, WA, USA). The obtained results were analyzed through SPSS Statistics software (IBM SPSS Statistics for Mac OS, Version 26.0; IBM Corp., Armonk, NY, USA). To determine if there were significant differences between samples, an analysis of variance (ANOVA) was also applied, while a comparison of the means was performed with the Student’s *t*-test (α = 0.05). Furthermore, a Pearson’s correlation analysis between the bioactivities and all the sum contents of the analyzed compounds (total phenolic acid, total flavonoids, and total phenolic compounds) was carried out, with a 95% confidence level.

A principal component analysis (PCA) was also performed in order to examine the contribution of each variable to the total diversity and classify the studied maturation stages according to their chemical compositions by using the statistical software Statgraphics 5.1.plus (Statpoint Technologies, Inc., Warrenton, VA, USA).

## 3. Results and Discussion

### 3.1. Phenolic Compounds Composition

**Figure 2:**
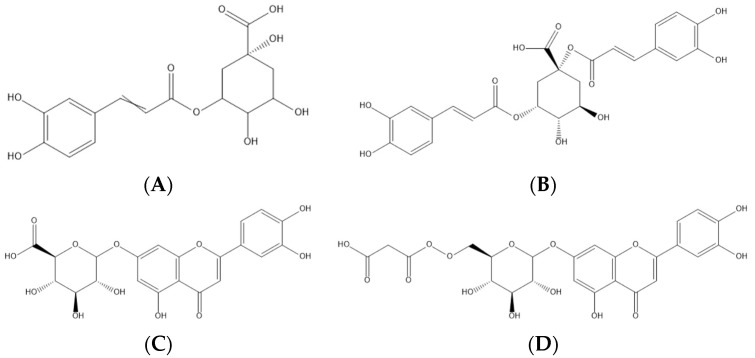
Representation of the four major phenolic compounds found in the cardoon samples studied—namely, two phenolic acids (5-*O*-caffeoylquinic—(**A**) and 1,5-di-*O*-caffeoylquinic acids—(**B**)) and two flavonoids (luteolin-*O*-hexuronoside—(**C**) and luteolin-*O*-malonyl-hexoside—(**D**)).

The results regarding the phenolic compound compositions, peak characteristics, and their tentative identifications are presented in Table 1. The quantification of each individual compound is presented in Table 2, as is the extraction yield (referring to the concentration of mg of compound/100 g of petioles but expressed in percentage) of each sample. In Figure 2 are presented the main phenolic acids and flavonoids found in the samples studied and, in the Supplementary Materials (SM1), the exemplified phenolic profiles of the sixteen samples of cardoon studied recorded at 280 nm. The phenolic compounds were tentatively identified according to their retention time (Rt), the wavelength of maximum absorbance (λ_max_), deprotonated ion ([M-H]^−^), and fragmentation pattern (MS^2^). A total of fifteen compounds were tentatively identified in cardoon petioles, including ten phenolic acid derivatives (peaks 1, 2, 3, 4, 5, 7, 10, 11, 12, and 15) and five flavonoid glycosides (peaks 6, 8, 9, 13, and 14).

Protocatechuic acid (peak 2) was identified by comparing the retention time and the maximum UV spectra with the chromatographic characteristics of the corresponding available standard compound. The caffeoylquinic and dicaffeoylquinic acids (peaks 1, 3, 4, 5, 7, 10, 11, 12, and 15) were identified according to the hierarchical keys proposed by Clifford et al. [31] and Clifford, Susan, and Nikolai [32], based on the deprotonated ion ([M-H]^−^ at *m*/*z* 353 and 515, respectively) and characteristic intensity of the fragment ions produced. The MSn spectra for the dicaffeoylquinic acids are provided in the Supplementary Materials (SM2). Regarding the flavonoids, peak 6 was tentatively identified as eriodictyol-*O*-hexuronoside, presenting a deprotonated ion [M-H]^−^ at *m*/*z* 463 and a unique MS^2^ fragment at *m*/*z* 287 (eriodictyol aglycone), corresponding to the loss of 176 u of a hexuronoside unit. Finally, two O-glycosylated luteolin derivatives were tentatively identified as luteolin-*O*-hexuronoside derivatives I and II (peaks 8 and 9, respectively), since both presented the same deprotonated ion [M-H]^−^ at *m*/*z* 461 and a unique MS^2^ fragment at *m*/*z* 285 (luteolin aglycone), corresponding to the loss of 176 u of a hexuronoside unit. The same behavior was observed for peaks 13 and 14, luteolin-*O*-malonyl hexoside derivatives I and II, respectively, presenting a deprotonated ion [M-H]^−^ at *m*/*z* 533 and MS^2^ fragments at *m*/*z* 489 and 447 (44 u + 42 u, malonyl group) and *m*/*z* 285 (162 u, a hexosyl moiety).

Peaks 1 and 3 (3-*O*-caffeoylquinic and 4-*O*-caffeoylquinic acids) have been previously described in cardoons [20,21,33,34]. Similarly, peaks 4 and 5 (*cis*-5-*O*-caffeoylquinic and *trans*-5-*O*-caffeoylquinic acids) were previously identified in different cardoon tissues [10,22], peak 6 (Eriodictyol-*O*-hexuronoside) in cardoon inflorescences [21] and bracts [10], and peaks 8 and 9 in cardoon heads [22] and bracts [10]. Peaks 13 and 14 have been previously described in cardoon bracts [10] and inflorescences [21]. Different isomers of dicaffeoylquinic acids have also been reported in different cardoon tissues, such as *trans*-3,4-*O*-dicaffeoylquinic, *cis*-3,5-di-*O*-caffeoylquinic, and *trans-*3,5-di-*O*-caffeoylquinic acids [33], as also in cardoon leaf midribs and petioles (*trans*-4,5-di-*O*-caffeoylquinic acid) [33]. Finally, peak **2** was tentatively identified as protocatechuic acid based on the chromatographic information described by Graça et al. [35] and its previous detection in the bracts of *Cynara cardunculus* var. *scolymus* [36]. To the best of our knowledge, protocatechuic acid has not yet been reported in the *altilis* variety.

5-*O*-Caffeoylquinic (peak 3) and 1,5-*O*-Dicafffeoylquinic acids (peak 11) were the phenolic compounds present in higher abundance throughout the studied maturation stages (6.1–37.2 and 5.03–30.1-mg/g extract, respectively) (Table 2). Samples of immature petioles (samples P1–4) are those at which higher contents of phenolic compounds were determined (69.7–101-mg/g extract), especially sample P3 (101-mg/g extract), which refers to the early stage of PGS 2. Many of the identified compounds were not found in the petiole samples harvested at the senescence (samples P15 and P16) and the early growth stages (samples P1–3). Only six of the identified phenolic compounds were detected in these samples (mostly derived from caffeoylquinic and dicaffeoylquinic acids). Moreover, differences in the extraction yields were observed between the tested samples (Table 2), with samples at the late maturity stages (P12–P16) showing the lowest extraction yield. This finding could be associated with the lignification that takes places at late maturity, which could make less effective the tested protocols in the polyphenol extractions [37,38]. Therefore, the results of this study showed that harvesting time has an influence on the phenolic content and composition in petioles. Although several reports have already proven that the stage of maturity influences the phenolic composition of different plant tissues of cardoons (flower heads, bracts, and receptacle), to the best of our knowledge, none of these studies has analyzed the phenolic composition of the petioles throughout the growth cycle.

Mandim et al. [10] analyzed cardoon bracts harvested in different maturation stages, identifying twelve phenolic compounds, with 3,5-di-*O*-caffeoylquinic acid and apigenin-7-*O*-glucuronide as the most abundant ones and immature bracts showing the highest content of total phenolics (29.73-mg/g extract). Additionally, the phenolic composition of cardoon capitulum varies throughout the plant’s growth cycle, with the youngest tissues presenting higher amounts of phenolic compounds (34.3-mg/g extract) [22]. By contrast, the receptacle showed an increase in the contents of phenolic compounds in the months of February, March, and April [39]. It was suggested that the variation in the phenolic content is related to the translocation of the compounds to parts of the plant with the highest biosynthetic activity at a given time [22]. Additionally, aspects such as growing location and conditions, variety, and post-harvest storage have an influence on the contents of phenolic compounds [21,30,33]. The study of the influence of all these factors is extremely useful and important for the proper use and the economic value of the species. The obtained results could contribute useful information regarding the valorization of the cardoon petioles, which represent a high portion of plant biomass throughout its growth cycle. Therefore, harvests at specific growth stages could allow the obtainment of extracts with a high content in targeted compounds that could be used for pharmaceutical and nutraceutical purposes or in the food industry for the production of healthy and functional foods.

### 3.2. Bioactive Properties

#### 3.2.1. Antioxidant Potential

The antioxidant activity of the hydroethanolic extracts of cardoon petioles was studied using two cell-based methodologies (TBARS and OxHLIA), and the obtained results are presented in Table 3. All the analyzed samples exhibited the ability to inhibit the oxidation process in both cell-based assays performed. For the TBARS assay, the samples in the early maturation stages revealed, in general, lower IC_50_ values (i.e., more potent antioxidant activity); in particular, sample P3 (PSG 1), with the highest phenolic content among the analyzed samples, showed the highest antioxidant activity, with an IC_50_ value lower than the positive control Trolox (IC_50_ value of sample P3: 5.0 µg/mL; Trolox: 9.1 µg/mL).

On the other hand, sample P4, also at early maturity (PGS 2), was the most effective to protect the erythrocytes from hemolysis (OxHLIA assay), showing the lowest IC_50_ values compared with the remaining samples (IC_50_ values of 65 and 180 µg/mL for Δ*t* of 60 and 120 min, respectively). In contrast, the extracts of P15 (PSG 8/9) for TBARS and the P2 and P3 (PSG 1) samples for OxHLIA were the ones with the highest IC_50_ values and, therefore, with the lowest antioxidant activity.

The antioxidant potential in cardoon samples is one of the most-studied and described bioactivities of the species, mainly using the radical-scavenging capacity through the DPPH assay. The studies described in the literature verified that the antioxidant potential associated with this species depends on parameters such as the type and viability of the plant tissue, the genetic information, and the maturity state [10,21,33,40]. In the present study, the better antioxidant results in the TBARS assay were obtained in the samples at early maturity coinciding with the highest content of phenolic compounds. With the correlation study performed, it was possible to observe a negative correlation between the phenolic content and both antioxidant activity assays performed, since the higher the concentration of phenolic compounds, the lower the IC_50_ values (and, therefore, greater antioxidant activity). However, the *R* values were not very satisfactory, since phenolic acids (*R* = −0.296), flavonoids (*R* = −0.260), and total compounds (*R* = −0.301) only explain, approximately, 10% of the values obtained in the TBARS assay. In previous studies, a negative correlation was confirmed between the total phenolic compounds and phenolic acid content and high antioxidant activity as well [41,42]. Moreover, according to the study of Pagano et al. [43], the antioxidant activities observed were mostly correlated with the dicaffeoylquinic acid contents (−0.93 to −0.98 values for the Pearson’s coefficient), whereas lower coefficient values (−0.5) were observed for the caffeoylquinic acids. This finding could partly explain the findings of our study, where caffeoylquinic acids were the most abundant phenolic compounds. However, the same was not verified for the OxHLIA assay, pointing to other compounds that could be involved in the antioxidant capacity as evaluated by this method, e.g., sesquiterpene lactones and inulin [44,45]. According to the literature, the results regarding the correlation between the phenolic compound contents and the antioxidant activity are contradictory and can be highly affected by the extraction protocols and other parameters related to the genetic material and growing conditions [32,46] or the plant part [33,38]. Moreover, the fact that this study evaluates the antioxidant potential of petioles throughout the growth cycle is maybe another reason that our results are in contrast with other reports and show weak correlations between the phenolic compounds and antioxidant activity. Considering the variable chemical compositions during the growth season, the correlation analysis did not allow us to obtain as high Pearson’s coefficient values, as in the literature reports where a single harvest [33,41,42,43] or a limited number of harvests were applied [10,22].

To the best of the authors’ knowledge, this is the first report regarding the antioxidant activity of cardoon petioles collected throughout the growth cycle. In general, cardoon petioles exhibited higher antioxidant potential than cardoon bracts and heads with the same genetic information previously studied by our group [10,40].

#### 3.2.2. Anti-Inflammatory Activity

The evaluation of the anti-inflammatory activity was performed through the measurements of the capacity to inhibit the proinflammatory mediator NO by the LPS-stimulated murine macrophage cell line (RAW 264.7). The obtained results are presented in Table 4. All petiole extracts exhibited anti-inflammatory activity, except for samples P5, P6, and P7 (IC_50_ > 400 µg/mL). The anti-inflammatory activity of the cardoon petiole extracts varied over the entire growth cycle, which suggests that the growing stage has an influence on the anti-inflammatory potential. The sample collected at principal growth stage 5 (P9) revealed the highest anti-inflammatory capacity, with an IC_50_ value of 14.2 µg/mL, which was lower than the positive control dexamethasone (IC_50_ = 16 µg/mL). This finding further supports our previous study regarding the correlation of antioxidant activity and phenolic compound contents, since sample P9 did not contain the highest amount of any detected phenolic compound, thus implying the presence of other bioactive compounds not detected in our study.

Studies on the potential of cardoon as a source of compounds with anti-inflammatory power are scarce. Contrary to what was observed for the petiole extracts, immature samples of cardoon heads and bracts revealed the highest anti-inflammatory potential using the same cell-based assay: heads IC_50_ = 72 µg/mL [22] and bracts IC_50_ = 183 µg/mL [10]. The ability of cardoon seeds to inhibit the production of NO was also studied, and none of the extracts showed significant activity [40]. Petioles collected at principal growth stage 7/8 have an interesting potential to be explored as sources of compounds with anti-inflammatory potential, especially when considering that this stage refers to the late stages of maturity, where plants are usually harvested for other purposes. To the best of the authors’ knowledge, this is the first study that has assessed the anti-inflammatory potential of cardoon petioles throughout plant maturation.

#### 3.2.3. Cytotoxic Effects against Tumor and Nontumor Cells

The cytotoxic potential of cardoon petiole extracts is presented in Table 5. The results are expressed as the extract concentrations that cause 50% of the cell proliferation inhibition (GI_50_ values). According to the obtained results, the cytotoxic potential of the petiole extracts not only depends on the growth stage but, also, on the employed type of cell lines. HeLa is the cell line that presented the greatest susceptibility, with lower values of GI_50_ at almost all states of maturity, except for samples P1, P3, and P16 (Table 5). For these samples, the HepG2 and MCF-7 cell lines were the most susceptible ones (lowest GI_50_ values). Samples at the mid-to-late maturation stages (especially sample P9) had greater cytotoxic potential, in contrast to the samples harvested at the early growth stages (samples P4–8) that presented higher GI_50_ values. In general, the GI_50_ values for the nontumor cells (PLP2) were higher (i.e., lower cytotoxicity) than those obtained for the human tumor cell lines. Considering our findings regarding the anti-inflammatory activity where sample P9 recorded the best performance among the tested samples, bioactive compounds other than polyphenols should be acclaimed for the observed activities.

The study of the influence of the maturation stage on the cytotoxic potential of *Cynara cardunculus* plant tissues has previously been studied in samples of bracts [10] and the capitula (heads) [22]. In both studies, the younger stages of maturation showed greater cytotoxic, contrary to what was observed for the petiole extracts. As previously noticed [21,40], in addition to the stage of maturity and plant tissue, the bioactive potential may also be influenced by factors such as the genetic information, growing location, and tissue viability. To the best of the authors’ knowledge, the influence of the maturity stage on the cytotoxic potential of the cardoon petioles has not yet been described in the literature.

#### 3.2.4. Antimicrobial Activity

The results obtained in the antibacterial assessment of the cardoon petioles are presented in Table 6. All the tested extracts revealed the capacity to inhibit the bacterial growth; in general, the Gram-positive bacteria revealed a higher susceptibility than the Gram-negative. These results are in agreement with previous reports, due to a greater susceptibility of Gram-positive bacteria as a result of their membrane constitution [10,22,47]. In general, petioles at principal growth stage 3 (sample P5) showed higher antibacterial activity, with lower MIC values for all the bacteria tested (MIC values between 0.75 and 1.51 mg/mL), even though the effectiveness varied greatly in the different types of bacteria studied. While the early maturation states (samples P2 and P3) showed a lower potential against the bacteria *Bacillus cereus*, *Escherichia coli*, and *Salmonella* Typhimurium (MIC values between 2.31 and 4.78 mg/mL), sample P4 (PSG 2) was the least effective against *Staphylococcus aureus* (MIC values of 6.84 mg/mL), sample P16 showed a low effectiveness against *Listeria monocytogenes* (MIC values of 4.73 mg/mL), and samples P11–13 were the least effective against *Enterobacter cloacae* (MIC values between 3.24 and 3.63 mg/mL). Sample P7 (PSG 3/4) also demonstrated an interesting effectiveness against *Escherichia coli* and *Salmonella* Typhimurium (MIC values between 0.78 and 1.57 mg/mL). Gram-positive *Bacillus cereus* was the more susceptible bacteria (MIC values between 0.75 and 2.39 mg/mL). On the other hand, *Staphylococcus aureus* was the bacteria that revealed higher MIC values and, therefore, lower susceptibility (MIC values between 1.51 and 6.84 mg/mL). Nevertheless, none of the tested extracts presented higher activity than the positive controls used (i.e., commercial antibiotics streptomycin and ampicillin).

Cardoon petioles presented higher antibacterial activity than other plant tissues of cardoons previously studied, namely heads [22], bracts [10], viable and nonviable seeds harvested in Viseu, Portugal [40], and inflorescences from different genotypes [21]. These results further evidenced the influence that the different plant tissues, genetic information, growing location, and maturation stage of the species may have on its bioactive potential. Moreover, as mentioned in the case of the anti-inflammatory and cytotoxic activities, the phenolic profiles of the best-performing samples (P2 and P5) did not justify a correlation between the phenolic compound contents and the antibacterial activities of the cardoon petioles, and other bioactive compounds should be implicated.

The antifungal potential of the cardoon petioles collected at different maturation states was also analyzed, and the results are presented in Table 7. The antifungal potential changed depending on the fungi tested. In general, samples with mid-to-late maturations stages were more effective—namely, samples P10 and P11 against the fungi strains *Penicillium funiculosum*, *Penicillium ochrochloron*, and *Penicillium verrucosum* var. *cyclopium* (MIC values between 0.30 and 0.91 mg/mL). Sample P10 (PSG 5/6) presented lower MIC values than the positive control used (commercial antifungal ketoconazole). Sample P14 showed a higher efficiency against *Aspergillus fumigatus* (MIC value of 0.28 mg/mL). The sample collected at early maturity (sample P5) was the one with the highest antifungal capacity against *Aspergillus versicolor* (MIC value of 0.50 mg/mL). On the other hand, petioles harvested at the early maturation stages (samples P1–3) had less antifungal potential, particularly against *Aspergillus fumigatus*, *Aspergillus versicolor*, and *Penicillium ochrochloron* (MIC values between 0.92 and 3.71 mg/mL). For the remaining tested fungi strains (*Aspergillus niger*, *Penicillium funiculosum*, and *Penicillium verrucosum* var. *cyclopium*), petioles at lower maturity levels (samples P2–P5) revealed the highest MIC values and, therefore, the lowest antifungal potential. Previous works have already proven the antifungal potential of cardoon. It was verified that the maturity stage of cardoon heads and bracts had an influence on the antifungal potential [10,22] but, also, the genetic information [21] and the plant tissue [10,22,40]. The variable activities of the studied samples against the tested fungi imply that bioactive compounds other than polyphenols should be implicated in the observed antifungal properties of cardoon petioles.

### 3.3. Principal Component Analysis (PCA)

**Figure 3:**
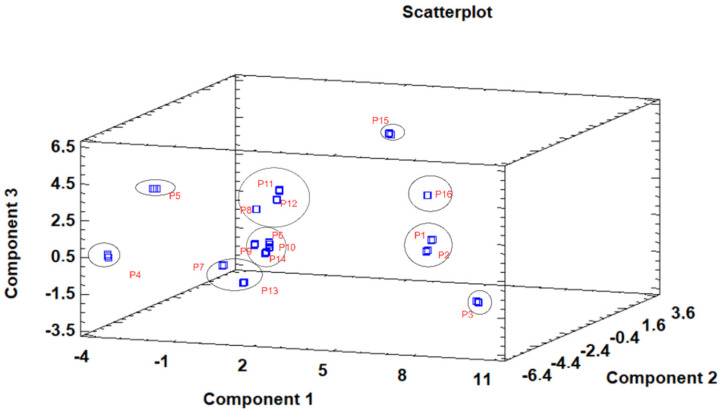
Three−dimensional principal component scatterplot of the tested variables at different maturation stages of cardoon petioles (samples P 1–16).

**Figure 4:**
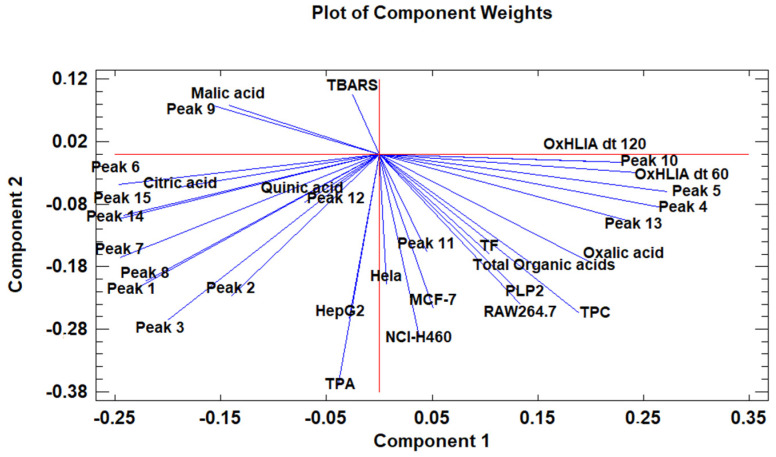
The principal component loading plot of the tested variables at different maturation stages of cardoon petioles.

The principal component analysis (PCA) is used to reduce the complexity of multivariate data as a means of identifying specific patterns and expressing data in ways that highlight similarities and differences and, further, visualize groups of samples according to their maturation stage [48,49]. The first seven principal components (PCs) were associated with Eigen values higher than 1 and explained 91.74% of the cumulative variance, with PC1 accounting for 39.51%, PC2 for 17.62%, PC3 for 11.91%, PC4 for 7.39%, PC5 for 6.71%, PC6 for 4.54%, and finally, PC7 for 4.06%. For simplification reasons, only the first three PCAs will be considered, since they added more than 10% to the cumulative variance, up to a total of 66.23%. PC1 was positively correlated with phenolic compounds with peak numbers **4**, **5**, **6**, and **13** and total phenolic compounds PLP2 and OxHLIA Δt60 and negatively correlated with TBARS and phenolic compounds with peak numbers **7**, **8**, **14**, and **15** and malic and citric acids. PC2 was only negatively correlated with the phenolic compound with peak number **3**, total phenolic acids, total phenolic compounds, HepG2, MCF-7, RAW264.7, PLP2, oxalic acid, quinic acid, and total organic acid. PC3 was positively correlated with phenolic compounds with peak numbers **2** and **3**, HepG2, NCI-H460, and quinic acids and negatively correlated with phenolic compounds with peak numbers **9** and **12** and the total flavonoids. These results indicated the correct application of the PCA, allowing the differentiation of petiole samples between the tested maturity stages, as shown in the corresponding scatterplot (Figure 3). Moreover, the presented plot suggests that the differences in the chemical compositions and bioactive properties of the tested petiole samples are correlated with the maturation stage. In particular, nine distinct groups were detected consisting of samples P1 and P2; sample P3; sample P4; sample P5; samples P6, P9, P10, and P14; samples P7 and P13; samples P8, 11, and 12; sample P15; and sample P16. The early (samples P1–P5) and late maturity stages (samples P15 and P16) are scattered in the scatterplot, whereas the intermedium maturity stages (samples P6–P14) are closely located. The loading plot (Figure 4) of the first two components revealed groups of positively correlated variables—namely, the upper-left quadrant comprising malic acid and the phenolic compound with peak number **9**; the lower-left quadrant comprising phenolic compounds with peak numbers **1**, **3**, **6**, **7**, **8**, **14**, and **15**; TPA; and citric acid; and the lower-right quadrant comprising phenolic compounds with peak numbers **4**, **5**, **10**, and **13**; oxalic acid; TPC; RAW264.7; NCI-H460; OxHLIA Δt60; and OxHLIA Δt120.

## 4. Conclusions

According to the obtained results, the phenolic compositions and biological properties of the cardoon petioles were influenced by the maturation stage. The samples at the early maturation stages (sample P3) revealed higher levels of phenolic compounds, especially 5-*O*-caffeoylquinic and 1,5-*O*-dicafffeoylquinic acids, which were detected in higher abundance. The sample P3 collected at early maturity also exhibited the highest capacity to inhibit the lipid peroxidation through TBARS formation inhibition, which could be attributed to the overall amounts of the phenolic compounds and/or the high contents in the 5-*O*-caffeoylquinic and 1,5-*O*-dicafffeoylquinic acids. In contrast, the samples at the mid-to-late maturation stages revealed the highest anti-inflammatory (sample P13) and cytotoxic (samples P7–P13) potential. A sample collected at the early stages (sample P4) exhibited the higher capacity to inhibit oxidative hemolysis and the bacterial growth, which could be associated with the highest content in total phenolic acids and 4-*O*-caffeoylquinic acid in particular. Regarding the antifungal potential, the tested extracts exhibited distinct behaviors against the fungi strains tested; while the samples at the intermediate maturation stages presented higher potential against *Penicillium funiculosum*, *Penicillium ochrochloron*, *Penicillium verrucosum* var. *cyclopium*, and *Aspergillus fumigatus*, the immature sample P5 was the most effective against *Aspergillus versicolor*. The fact that the samples at intermediate maturity did not contain high amounts of polyphenols indicates that other compounds not detected in the present study could be responsible for these activities. Considering that cardoon petioles are one of the less-explored and used tissues of this plant, collecting these valuable plant tissues at the proper maturity stages could help to obtain natural matrices with specific bioactive compounds and corresponding end uses. Moreover, an in-depth characterization in terms of the phenolic composition and bioactive properties will further contribute to the proper valorization of the species and its exploitation by different sectors. In conclusion, the association of the observed bioactive properties with individual compounds could allow harvests at specific growth stages aiming to obtain extracts with high contents in targeted compounds that could be used in the pharmaceutical, nutraceutical, or in the food industries.

## Figures and Tables

**Table 1:** Phenolic compounds tentatively identified in the hydroethanolic extracts of cardoon petioles.

Peak	Rt (min)	λ_max_ (nm)	[M-H]^−^ (*m*/*z*)	MS^2^ (*m*/*z*)	Tentative Identification
1	4.18	321	353	191 (100), 179 (33), 173 (5), 135 (5)	3-*O*-Caffeoylquinic acid
2	6.14	266	153	109 (100)	Protocatechuic acid
3	6.52	321	353	173 (100), 179 (11), 191 (10), 161 (5), 135 (5)	4-*O*-Caffeoylquinic acid
4	6.63	326	353	191 (100), 179 (7), 173 (5), 135 (5)	*cis*-5-*O*-Caffeoylquinic acid
5	7.10	326	353	191 (100), 179 (7), 173 (5), 135 (5)	*trans*-5-*O*-Caffeoylquinic acid
6	15.97	285/sh324	463	287 (100)	Eriodictyol-*O*-hexuronoside
7	16.69	322	515	353 (100), 335 (25), 191 (62), 179 (15)	1,3-di-*O*-caffeoylquinic acid
8	18.61	266/343	461	285 (100)	Luteolin-*O*-hexuronoside derivative I
9	18.86	267/343	461	285 (100)	Luteolin-*O*-hexuronoside derivative II
10	19.01	334	515	353 (100), 179 (10), 173 (29), 353 (10), 191 (10), 135 (8), 161 (5)	*O*-Dicaffeyolquinic acid
11	20.39	324	515	353 (100), 191 (12), 335 (10)	1,5-di-*O*-cafffeoylquinic acid
12	22.66	329	515	353 (100), 335 (5), 229 (2), 255 (2), 203 (2), 191 (75), 179 (13), 173 (5, MS^3^ base peak)	3,4-di-*O*-cafffeoylquinic acid
13	23.69	268/332	533	489 (100), 285 (20)	Luteolin-*O*-malonyl hexoside derivative I
14	23.77	267/346	533	285 (100), 489 (50), 447 (5)	Luteolin-*O*-malonyl-hexoside derivative II
15	25.58	330	515	353 (100), 191 (12, MS^3^ base peak)	3,5-di-*O*-caffeolyquinic acid

**Table 2:** Content of the phenolic compounds determined in the hydroethanolic extracts of cardoon petioles.

Peak	Quantification (mg Equivalents of the Corresponding Standard Used for Quantification Per g of Extract)															
	P1	P2	P3	P4	P5	P6	P7	P8	P9	P10	P11	P12	P13	P14	P15	P16
1	n.d.	n.d.	n.d.	1.22 ± 0.03 ^a^	0.83 ± 0.01 ^b^	0.52 ± 0.01 ^ef^	0.48 ± 0.02 ^g^	0.35 ± 0.01 ^i^	0.51 ± 0.01 ^f^	0.568 ± 0.002 ^c^	0.32 ± 0.01 j	0.42 ± 0.02 ^h^	0.559 ± 0.004 ^cd^	0.54 ± 0.01 ^de^	n.d.	n.d.
2	n.d.	n.d.	n.d.	1.38 ± 0.01 ^b^	2.7 ± 0.1 ^a^	1.22 ± 0.02 ^c^	0.42 ± 0.01 ^d^	0.290 ± 0.002 ^e^	0.32 ± 0.01 ^e^	0.297 ± 0.005 ^e^	0.342 ± 0.001 ^e^	0.064 ± 0.003 ^f^	0.279 ± 0.002 ^e^	0.058 ± 0.001 ^fg^	n.d.	n.d.
3	n.d.	n.d.	n.d.	37.2 ± 0.3 ^a^	33.9 ± 0.3 ^b^	12.18 ± 0.05 ^fg^	24.9 ± 0.4 ^c^	19.2 ± 0.1 ^d^	16.4 ± 0.2 ^e^	11.64 ± 0.02 ^h^	11.9 ± 0.2 ^gh^	6.1 ± 0.1 ^k^	8.7 ± 0.2 ^j^	9.7 ± 0.1 ^i^	n.d.	n.d.
4	3.6 ± 0.1 ^b^	3.70 ± 0.03 ^b^	5.4 ± 0.2 ^a^	n.d.	n.d.	n.d.	n.d.	n.d.	n.d.	n.d.	n.d.	n.d.	n.d.	n.d.	1.32 ± 0.03 ^d^	2.02 ± 0.1 ^c^
5	8.0 ± 0.2 ^c^	8.65 ± 0.05 ^b^	16.0 ± 0.5 ^a^	n.d.	n.d.	n.d.	n.d.	n.d.	n.d.	n.d.	n.d.	n.d.	n.d.	n.d.	2.524 ± 0.003 ^e^	4.17 ± 0.04 ^d^
6	n.d.	n.d.	n.d.	0.450 ± 0.001 ^d^	0.4143 ± 0.0001 ^g^	0.398 ± 0.001 ^h^	0.598 ± 0.04 ^a^	0.50 ± 0.01 ^e^	0.451 ± 0.001 ^d^	0.54 ± 0.01 ^b^	0.43 ± 0.01 ^f^	0.42 ± 0.01 ^fg^	0.44 ± 0.01 ^r^	0.444 ± 0.004 ^de^	n.d.	n.d.
7	n.d.	n.d.	n.d.	0.80 ± 0.01 ^a^	0.72 ± 0.01 ^b^	0.61 ± 0.01 ^de^	0.62 ± 0.01 ^cd^	0.393 ± 0.003 ^g^	0.60 ± 0.01 ^e^	0.457 ± 0.004 ^f^	0.348 ± 0.004 ^i^	0.313 ± 0.005 ^j^	0.631 ± 0.002 ^c^	0.38 ± 0.01 ^h^	n.d.	n.d.
8	n.d.	n.d.	n.d.	1.58 ± 0.01 ^a^	0.68 ± 0.02 ^e^	0.69 ± 0.01 ^e^	0.95 ± 0.01 ^b^	0.89 ± 0.03 ^c^	0.90 ± 0.03 ^c^	0.756 ± 0.002 ^d^	0.439 ± 0.001 ^h^	0.589 ± 0.001 ^f^	0.54 ± 0.01 ^g^	0.5411 ± 0.0004 ^g^	n.d.	n.d.
9	n.d.	n.d.	n.d.	0.471 ± 0.001 ^gh^	0.41 ± 0.01 ^hi^	0.395 ± 0.004 ^i^	0.96 ± 0.05 ^e^	0.49 ± 0.01 ^g^	1.10 ± 0.02 ^d^	1.26 ± 0.05 ^c^	0.75 ± 0.01 ^f^	0.76 ± 0.02 ^f^	3.49 ± 0.02 ^a^	2.6 ± 0.1 ^b^	n.d.	n.d.
10	3.6 ± 0.1 ^a^	1.29 ± 0.03 ^d^	1.71 ± 0.02 ^b^	n.d.	n.d.	n.d.	n.d.	n.d.	n.d.	n.d.	n.d.	n.d.	n.d.	n.d.	1.02 ± 0.04 ^e^	1.5 ± 0.1 ^c^
11	30.1 ± 0.4 ^a^	15.0 ± 0.1 ^e^	13.1 ± 0.4 ^g^	23.7 ± 0.4 ^b^	8.4 ± 0.3 ^j^	11.5 ± 0.3 ^h^	16 ± 1 ^d^	5.0 ± 0.1 ^l^	12.9 ± 0.3 ^g^	14.78 ± 0.02 ^ef^	6.9 ± 0.2 ^k^	5.42 ± 0.05 ^l^	22 ± 1 ^c^	13.1 ± 0.1 ^g^	9.6 ± 0.2^i^	14.25 ± 0.03 ^f^
12	2.4 ± 0.1 ^h^	3.2 ± 0.1 ^f^	3.5 ± 0.1 ^e^	4.58 ± 0.02 ^c^	1.63 ± 0.03 ^k^	2.8 ± 0.1 ^g^	3.03 ± 0.04 ^f^	1.69 ± 0.01 ^k^	3.6 ± 0.1 ^e^	4.4 ± 0.1 ^d^	1.60 ± 0.04 ^k^	2.2 ± 0.1 ^i^	6.3 ± 0.2 ^a^	5.30 ± 0.02 ^b^	1.54 ± 0.03^k^	1.90 ± 0.03 ^j^
13	1.48 ± 0.01 ^d^	3.05 ± 0.05 ^b^	10.8 ± 0.1 ^a^	n.d.	n.d.	n.d.	n.d.	n.d.	n.d.	n.d.	n.d.	n.d.	n.d.	n.d.	2.01 ± 0.04^c^	1.27 ± 0.02 ^e^
14	n.d.	n.d.	n.d.	1.08 ± 0.01 ^b^	0.70 ± 0.01 ^g^	0.62 ± 0.02 ^hi^	1.16 ± 0.01 ^a^	0.679 ± 0.002 ^g^	0.94 ± 0.01 ^c^	0.82 ± 0.04 ^e^	0.59 ± 0.02 ^i^	0.64 ± 0.02 ^h^	0.763 ± 0.002 ^f^	0.91 ± 0.02 ^d^	n.d.	n.d.
15	n.d.	n.d.	n.d.	0.79 ± 0.02 ^a^	0.457 ± 0.004 ^e^	0.54 ± 0.03 ^d^	0.38 ± 0.01 ^f^	0.29 ± 0.01 ^g^	0.63 ± 0.0 2 ^b^	0.45 ± 0.02 ^e^	0.4788 ± 0.0003 ^e^	0.30 ± 0.01 ^g^	0.7895 ± 0.0003 ^a^	0.57 ± 0.02 ^c^	n.d.	n.d.
TPA	47.8 ± 0.3 ^c^	31.8 ± 0.2 ^h^	40 ± 1 ^e^	69.7 ± 0. 1 ^a^	48.6 ± 0.4 ^b^	29.3 ± 0.5 ^i^	46.1 ± 0.4 ^d^	27.3 ± 0.2 ^j^	34.9 ± 0.4 ^f^	32.56 ± 0.04 ^g^	21.9 ± 0.4 ^l^	14.9 ± 0.2 ^n^	39.5 ± 0.2 ^e^	29.6 ± 0.2 ^i^	16.1 ± 0.2 ^m^	23.85 ± 0.03 ^l^
TF	1.48 ± 0.01 ^k^	3.05 ± 0.05 ^f^	10.8 ± 0.1 ^a^	3.59 ± 0.01 ^d^	2.211 ± 0.003 ^i^	2.10 ± 0.01 ^ij^	3.7 ± 0.1 ^d^	2.55 ± 0.05 ^g^	3.4 ± 0.1 ^e^	3.4 ± 0.1 ^e^	2.202 ± 0.005 ^i^	2.407 ± 0.001 ^h^	5.241 ± 0.003 ^b^	4.5 ± 0.1 ^c^	2.01 ± 0.04 ^j^	1.27 ± 0.02 ^l^
TPC	98.5 ± 0.6 ^b^	69.7 ± 0.3 ^d^	101 ± 1 ^a^	73.3 ± 0.1 ^c^	50.8 ± 0.4 ^e^	31.4 ± 0.5 ^k^	49.8 ± 0.3 ^f^	29.8 ± 0.1 ^l^	38.3 ± 0.3 ^h^	33.9 ± 0.1 ^i^	24.08 ± 0.39 ^m^	17.3 ± 0.2 ^n^	44.8 ± 0.2 ^g^	34.1 ± 0.3 ^j^	36.2 ± 0.5 ^l^	50.2 ± 0.1 ^ef^
Yield (%)	25.46	27.4	30.99	30.9	34.79	39.36	33.53	37.56	22.86	30.98	20.17	15.46	14.42	11.90	14.4	14.57

Results are presented as the mean ± standard deviation. Different letters in the same row correspond to significant differences (*p* < 0.05). n.d.: not detected. The information regarding the calibration curves used for quantification is described in Section 2.3. of the Material and Methods section. TPA: total phenolic acids (sum of the amounts of compounds **1**, **2**, **3**, **4**, **5**, **7**, **10**, **11**, **12**, and **15**); TF: total flavonoids (sum of the amounts of compounds **6**, **8**, **9**, **13**, and **14**); and TPC: total phenolic compounds (sum of the amounts of all fifteen compounds).

**Table 3:** Antioxidant activity of the hydroethanolic extracts of cardoon petioles.

Antioxidant Activity (IC_50_, µg/mL)			
Sample	TBARS	OxHLIA (Δ*t* = 60 min)	OxHLIA (Δ*t* = 120 min)
P1	15.8 ± 0.1 ^m^	244 ± 5 ^b^	323 ± 7 ^e^
P2	22.6 ± 0.4 ^j^	392 ± 10 ^a^	563 ± 17 ^a^
P3	5.0 ± 0.1 ^o^	386 ± 2 ^a^	542 ± 7 ^a^
P4	75.6 ± 0.5 ^d^	65 ± 4 ^i^	180 ± 3 ^h^
P5	61.0 ± 0.5 ^e^	110 ± 5 ^h^	245 ± 7 ^fg^
P6	20.3 ± 0.2 ^l^	195 ± 5 ^d^	382 ± 5 ^c^
P7	20.8 ± 0.5 ^kl^	224 ± 9 ^bc^	466 ± 18 ^b^
P8	56.6 ± 0.5 ^f^	168 ± 4 ^e^	370 ± 4 ^cd^
P9	92 ± 1 ^b^	122 ± 4 ^gh^	206 ± 4 ^gh^
P10	58 ± 2 ^f^	122 ± 4 ^gh^	206 ± 4 ^gh^
P11	83.9 ± 0.4 ^c^	157 ± 6 ^e^	289 ± 9 ^ef^
P12	34.5 ± 0.5 ^h^	135 ± 5 ^fg^	266 ± 4 ^f^
P13	27 ± 2 ^i^	114 ± 2 ^gh^	185 ± 4 ^h^
P14	44.9 ± 0.5 ^g^	102 ± 4 ^hj^	201 ± 5 ^gh^
P15	287 ± 2 ^a^	208 ± 14 ^cd^	400 ± 40 ^c^
P16	21.9 ± 0.4 ^jk^	150 ± 8 ^ef^	243 ± 4 ^ef^
Trolox	9.1 ± 0.3 ^n^	21.2 ± 0.7 ^k^	41.1 ± 0.8 ^i^

Results are expressed as the mean ± standard deviation. Different letters in the same column correspond to significant differences (*p* < 0.05). IC_50_ values correspond to the extract concentrations needed to inhibit 50% of the formation of thiobarbituric acid reactive substances (TBARS), the oxidative hemolysis (OxHLIA).

**Table 4:** Anti-inflammatory activity of the hydroethanolic extracts of cardoon petioles.

Anti-Inflammatory Activity (IC_50_; µg/mL)	
Sample	RAW 246.7
P1	154 ± 4 ^d^
P2	91 ± 2 ^e^
P3	179 ± 3 ^c^
P4	222 ± 13 ^a^
P5	>400
P6	>400
P7	>400
P8	191 ± 10 ^b^
P9	14.2 ± 0.5 ^h^
P10	34 ± 4 ^g^
P11	40 ± 2 ^g^
P12	36 ± 2 ^g^
P13	18 ± 2 ^h^
P14	31 ± 1 ^g^
P15	80 ± 3 ^f^
P16	79 ± 3 ^f^
Dexamethasone	16 ± 1 ^hi^

Results are expressed as the mean ± standard deviation. Different letters in the same column correspond to significant differences (*p* < 0.05). IC_50_ values correspond to the extract concentrations needed to inhibit 50% of the nitric oxide (NO) production.

**Table 5:** Cytotoxic activity of the hydroethanolic extracts of cardoon petioles.

Cytotoxic Activity (GI_50_; µg/mL)					
Sample	MCF-7	NCI-H460	HeLa	HepG2	PLP2
P1	150 ± 3 ^d^	173 ± 14 ^d^	153 ± 6 ^d^	155 ± 9 ^d^	239 ± 16 ^d^
P2	76 ± 1 ^e^	80 ± 6 ^e^	58 ± 5 ^g^	65 ± 4 ^e^	143 ± 7 ^d^
P3	191 ± 4 ^c^	223 ± 7 ^c^	141 ± 8 ^de^	68 ± 2 ^e^	336 ± 11 ^a^
P4	253 ± 5 ^b^	238 ± 23 ^c^	132 ± 5 ^e^	228 ± 9 ^c^	307 ± 9 ^b^
P5	>400	353 ± 25 ^a^	20 ± 2 ^c^	351 ± 14 ^a^	>400
P6	>400	>400	204 ± 15 ^a^	>400	314 ± 19 ^b^
P7	345 ± 20 ^a^	>400	269 ± 8 ^b^	>400	>400
P8	203 ± 15 ^c^	312 ± 8 ^b^	82 ± 3 ^f^	304 ± 11 ^b^	282 ± 12 ^c^
P9	12 ± 1 ^ij^	16.0 ± 0.5 ^hi^	11.1 ± 0.3 ^i^	14 ± 1 ^g^	16 ± 1 ^i^
P10	43 ± 2 ^h^	36 ± 2 ^gh^	17 ± 1 ^i^	17 ± 2 ^g^	49 ± 3 ^jk^
P11	58 ± 4 ^fg^	55 ± 1 ^fg^	38 ± 2 ^h^	62 ± 5 ^e^	61 ± 2 ^j^
P12	26 ± 1 ^i^	35 ± 1 ^gh^	19 ± 1 ^i^	16 ± 1 ^g^	44 ± 1 ^jk^
P13	23 ± 1 ^i^	70 ± 1 ^ef^	17 ± 1 ^i^	19 ± 2 ^g^	37 ± 1 ^h^
P14	51 ± 3 ^gh^	30 ± 2 ^h^	20 ± 1 ^i^	19 ± 2 ^g^	55 ± 1 ^j^
P15	66 ± 5 ^ef^	86 ± 3 ^e^	54 ± 3 ^gh^	47 ± 4 ^f^	112 ± 4 ^e^
P16	60 ± 2 ^fg^	72 ± 5 ^ef^	55 ± 3 ^gh^	39 ± 3 ^f^	116 ± 6 ^e^
Ellipticine	1.21 ± 0.02 ^k^	0.9 ± 0.1 ^j^	1.03 ± 0.09 ^j^	1.10 ± 0.09 ^h^	2.3 ± 0.2 ^j^

Results are expressed as the mean ± standard deviation. Different letters in the same column correspond to significant differences (*p* < 0.05). GI_50_ values correspond to the extract concentrations that cause 50% of the cell growth inhibition.

**Table 6:** Antibacterial activity of the hydroethanolic extracts of the cardoon petioles.

Antibacterial Activity (mg/mL)												
	*B. cereus*		*S. aureus*		*L. monocytogenes*		*E. cloacae*		*E. coli*		*S.* *Typhimurium*	
	MIC	MBC	MIC	MBC	MIC	MBC	MIC	MBC	MIC	MBC	MIC	MBC
P1	1.17	2.33	2.33	4.66	2.33	4.66	2.33	4.66	2.33	4.66	2.33	4.66
P2	2.39	4.78	4.78	9.55	4.78	9.55	1.15	2.31	2.39	4.78	4.78	9.55
P3	2.31	4.61	4.61	9.23	2.31	4.61	4.61	9.23	4.61	9.23	4.61	9.23
P4	1.71	3.42	6.84	6.84	3.42	6.84	1.71	3.42	1.71	3.42	3.42	6.84
P5	0.75	1.51	1.51	3.02	1.51	3.02	1.51	3.02	0.75	1.51	1.51	3.02
P6	1.69	3.37	3.37	6.75	1.69	6.75	3.37	6.75	1.69	3.37	6.75	>6.75
P7	1.57	3.13	3.13	6.27	1.57	3.13	1.57	3.13	0.78	1.57	1.57	3.13
P8	1.63	3.27	1.63	3.27	1.63	3.27	1.63	3.27	0.82	1.63	1.63	3.27
P9	1.89	3.78	1.89	3.78	1.89	3.78	1.89	3.78	0.94	1.89	1.89	3.78
P10	1.78	3.55	3.55	7.11	1.78	3.55	1.78	3.55	1.78	3.55	3.55	7.11
P11	1.81	1.81	3.63	7.26	3.63	7.26	3.63	7.26	1.81	3.63	3.63	7.26
P12	0.81	1.62	3.24	6.48	3.24	6.48	3.24	6.48	3.24	6.48	3.24	6.48
P13	1.72	1.72	3.43	3.43	3.43	6.87	3.43	6.87	3.43	6.87	3.43	6.87
P14	0.85	1.71	1.71	3.42	1.71	3.42	1.71	3.42	1.71	3.42	1.71	3.42
P15	1.15	2.31	2.31	4.61	2.31	4.61	2.31	4.61	2.31	4.61	2.31	4.61
P16	1.18	2.36	4.73	9.46	4.73	9.76	2.36	4.73	2.36	4.73	2.36	4.73
Streptomycin	0.10	0.20	0.04	0.10	0.20	0.30	0.20	0.30	0.20	0.30	0.20	0.30
Ampicillin	0.25	0.40	0.25	0.45	0.40	0.50	0.25	0.50	0.40	0.50	0.75	1.20

MIC: minimal inhibitory concentration; MBC: minimal bactericidal concentration. Positive control: streptomycin and ampicillin.

**Table 7:** Antifungal activity of the hydroethanolic extracts of cardoon petioles.

Antifungal Activity (mg/mL)												
	*A. fumigatus*		*A. versicolor*		*A. niger*		*P. funiculosum*		*P. ochrochloron*		*P. verrucosum* var. *cyclopium*	
	MIC	MFC	MIC	MFC	MIC	MFC	MIC	MFC	MIC	MFC	MIC	MFC
P1	3.71	7.42	1.86	3.71	1.86	3.71	0.93	1.86	1.86	3.71	1.86	3.71
P2	1.83	3.66	1.83	3.66	3.66	7.39	0.92	1.83	0.92	1.83	0.92	1.83
P3	3.71	7.42	0.93	1.86	1.86	3.71	0.93	1.86	3.71	7.42	1.86	3.71
P4	1.14	2.28	0.57	1.14	>9.12	> 9.12	0.57	1.14	0.57	1.14	0.57	1.14
P5	1.01	2.01	0.50	1.01	>8.05	>8.05	>8.05	>8.05	1.01	2.01	>8.05	>8.05
P6	1.12	2.25	1.12	2.25	>9	>9	>9	>9	5.62	1.12	9	>9
P7	0.52	1.04	0.52	1.04	>8.36	>8.36	0.52	1.04	0.52	1.04	1.04	2.09
P8	2.18	4.36	1.09	2.18	1.09	2.18	0.54	1.09	0.54	1.09	0.54	1.09
P9	2.52	5.04	1.26	2.52	1.26	2.52	1.26	2.52	1.26	2.52	0.63	1.26
P10	2.37	4.74	1.18	2.37	1.18	2.37	0.59	1.18	0.30	0.59	0.30	0.59
P11	0.91	1.81	0.91	1.81	1.81	3.63	0.45	0.91	0.91	1.81	0.45	0.91
P12	0.54	1.08	0.54	1.08	0.54	1.08	1.08	2.16	1.08	2.16	1.08	2.16
P13	0.57	1.14	1.14	2.29	1.14	2.29	0.57	1.14	0.57	1.14	0.57	1.14
P14	0.28	0.57	0.57	1.14	1.14	2.28	0.57	1.14	1.14	2.28	0.57	2.28
P15	0.90	1.80	1.80	3.60	3.60	7.18	1.80	3.60	0.90	1.80	1.80	3.60
P16	3.64	7.28	0.91	1.82	1.82	3.64	0.91	1.82	1.82	3.64	0.91	1.82
Ketoconazole	0.25	0.50	0.20	0.50	0.20	0.50	0.20	0.50	1.00	1.50	0.20	0.30

MIC: minimal inhibitory concentration; MFC: minimal fungicidal concentration. Positive control: ketoconazole.

## Data Availability

The data is contained within the article and the Supplementary Materials.

## Supplementary Materials

The following are available online at www.mdpi.com/article/10.3390/antiox10121907/s1: SM1. Exemplificative phenolic profiles of the sixteen samples of cardoons studied recorded at 280 nm. SM2. MS^2^ (1) and MS^3^ (2) spectra of the dicaffeoylquinic acid isomers found in petioles samples of cardoon: A1/A2-1,3-di-*O*-caffeoylquinic acid, B1/B2: *O*-dicaffeoylquinic acid, C1/C2: 1,5-di-*O*-cafffeoylquinic acid, D1/D2: 3,4-di-*O*-cafffeoylquinic acid, and E1/E2: 3,5-di-*O*-caffeolyquinic acid.
