# Supplementary material for: Phenolic Composition and Biological Properties of Cynara cardunculus L. var. altilis Petioles: Influence of the Maturity Stage
Source: Antioxidants (Basel). 2021 Nov 28;10(12):1907. doi: 10.3390/antiox10121907 (PMC8750300; doi:10.3390/antiox10121907)
Supplement: Supplementary file 1 [file antioxidants-10-01907-s001.zip › antioxidants-1474997-Supplementary.pdf]

Supplementary material

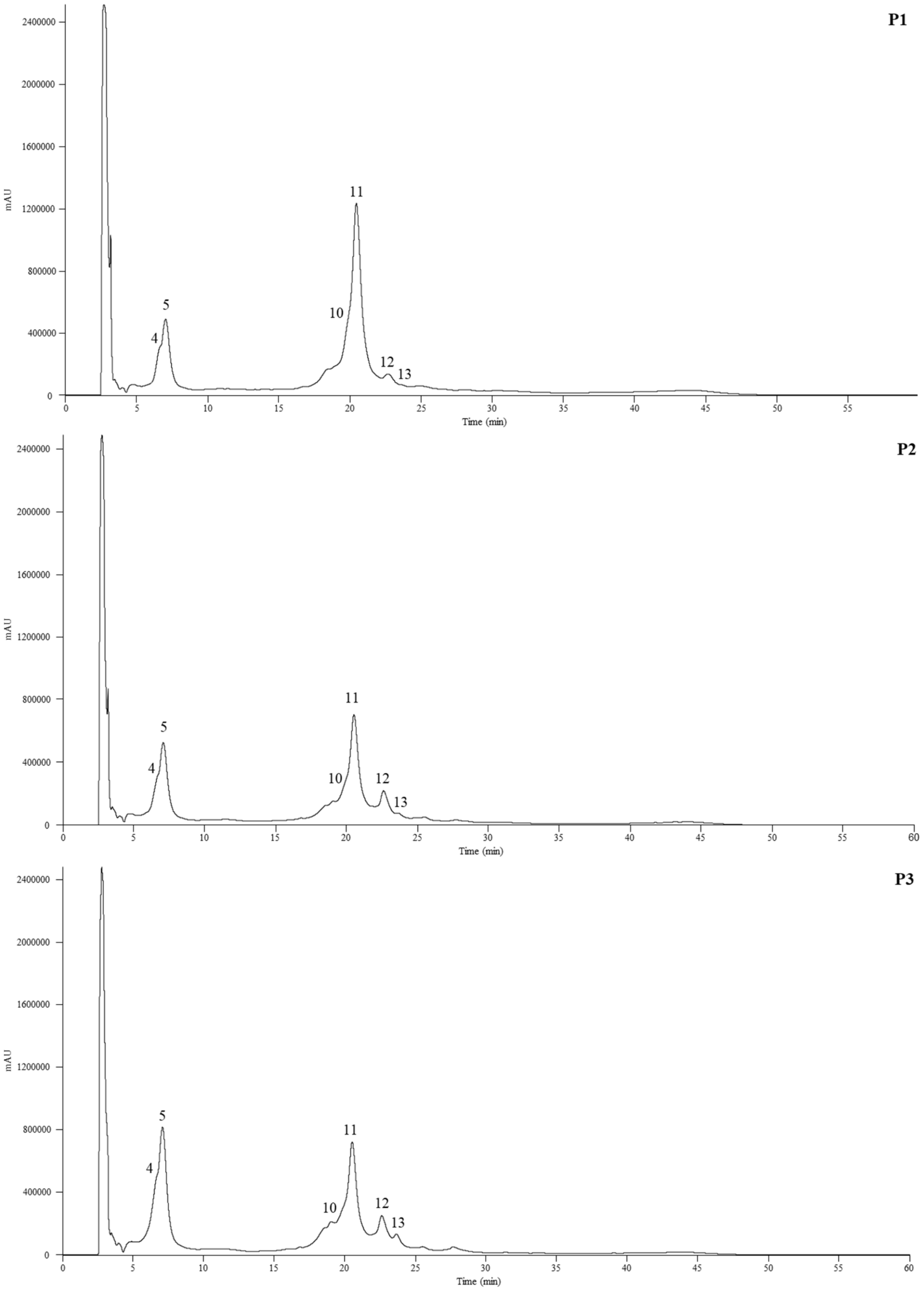

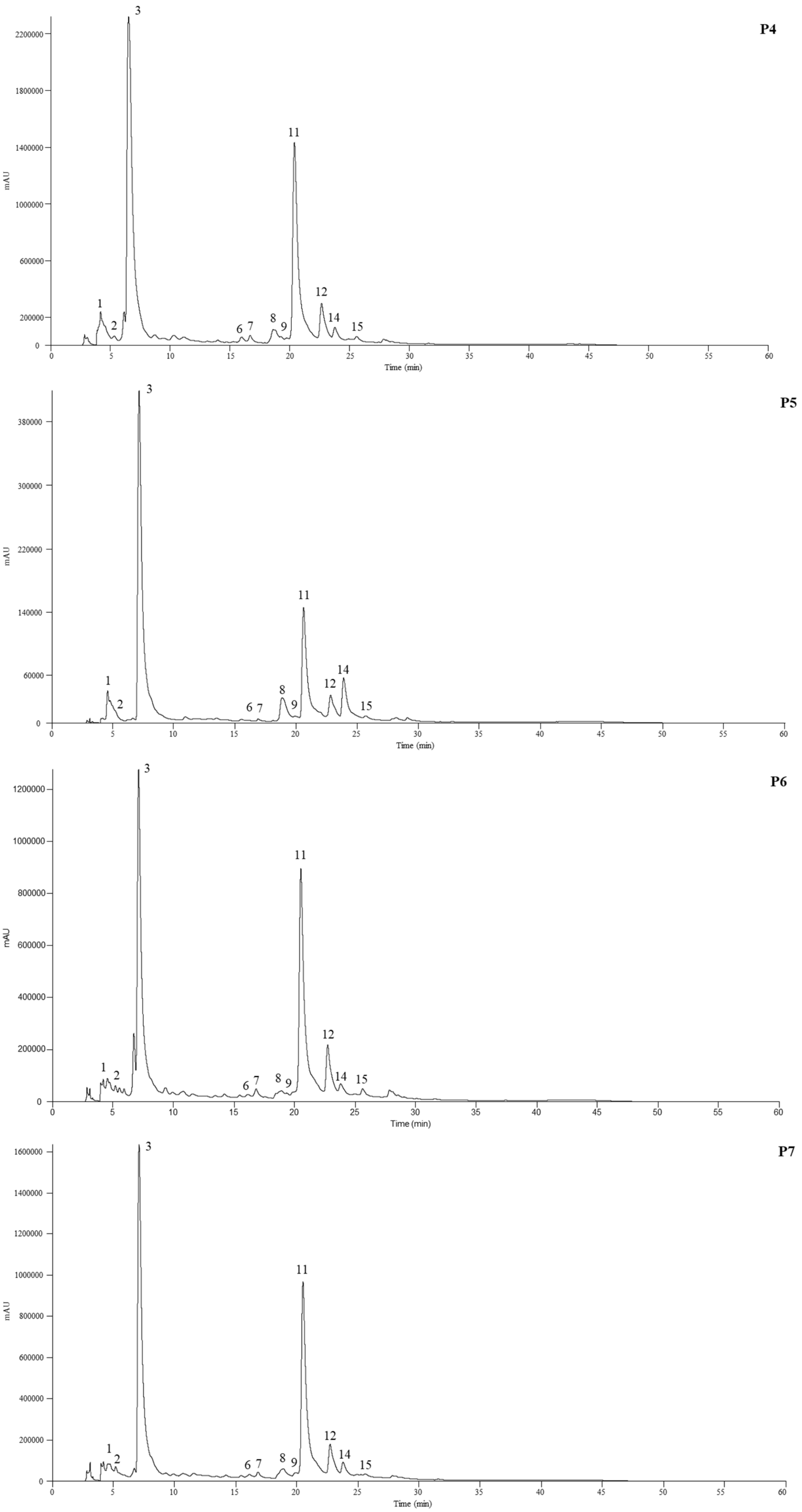
P4
mAU
Time (min)
0
200000
600000
1000000
1400000
1800000
2200000
0 5 10 15 20 25 30 35 40 45 50 55 60
1 2 3 6 7 8 9 11 12 14 15
P5
mAU
Time (min)
0
60000
140000
220000
300000
380000
0 5 10 15 20 25 30 35 40 45 50 55 60
1 2 3 6 7 8 9 11 12 14 15
P6
mAU
Time (min)
0
200000
400000
600000
800000
1000000
1200000
0 5 10 15 20 25 30 35 40 45 50 55 60
1 2 3 6 7 8 9 11 12 14 15
P7
mAU
Time (min)
0
200000
400000
600000
800000
1000000
1200000
1400000
1600000
0 5 10 15 20 25 30 35 40 45 50 55 60
1 2 3 6 7 8 9 11 12 14 15

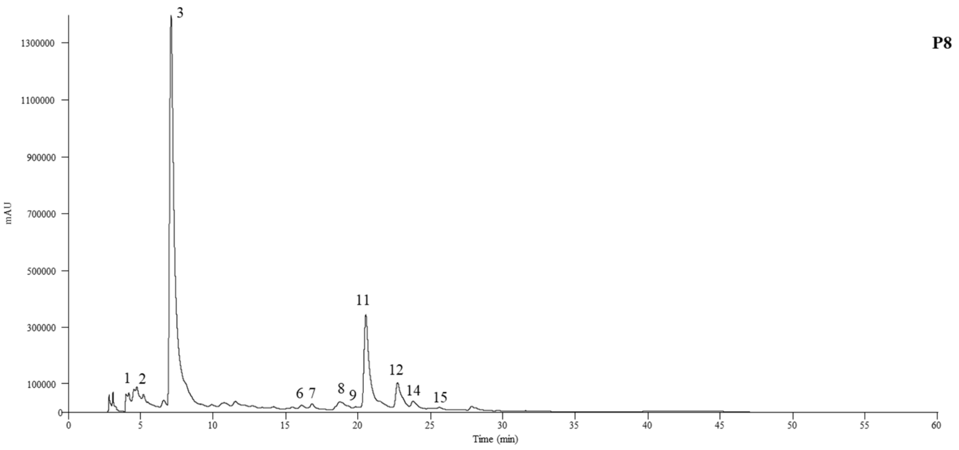
P8
mAU
Time (min)
1
2
3
6
7
8
9
11
12
14
15

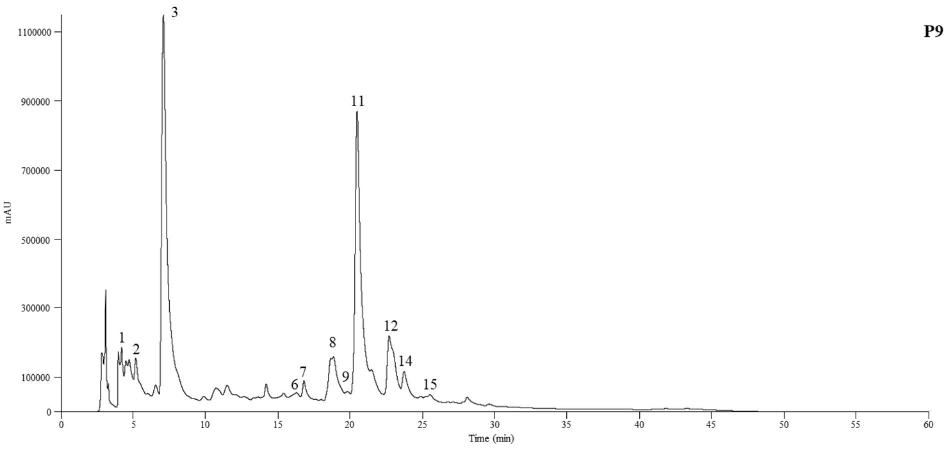
P9
mAU
Time (min)
1
2
3
6
7
8
9
11
12
14
15

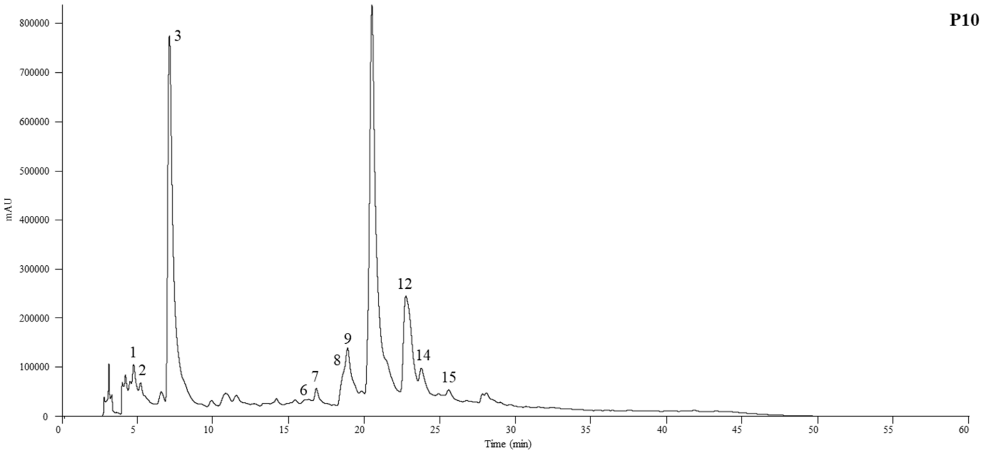
P10
mAU
Time (min)
1
2
3
6
7
8
9
12
14
15

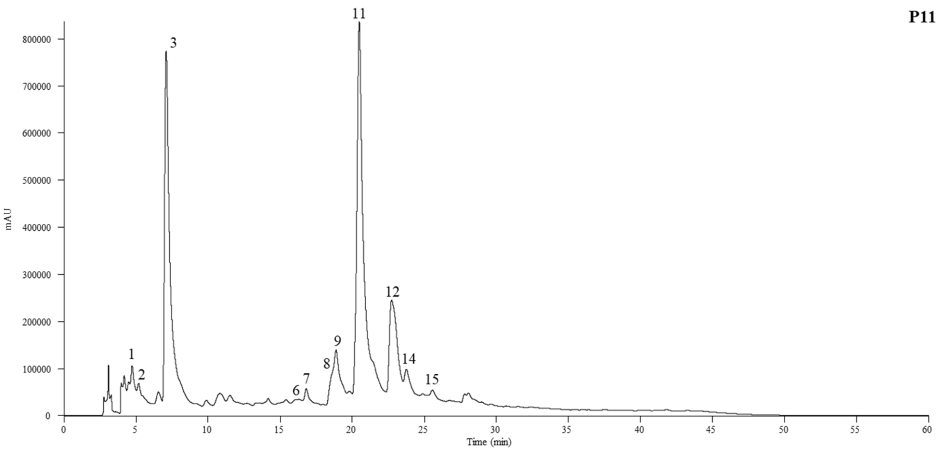
P11
mAU
Time (min)
1
2
3
6
7
8
9
11
12
14
15

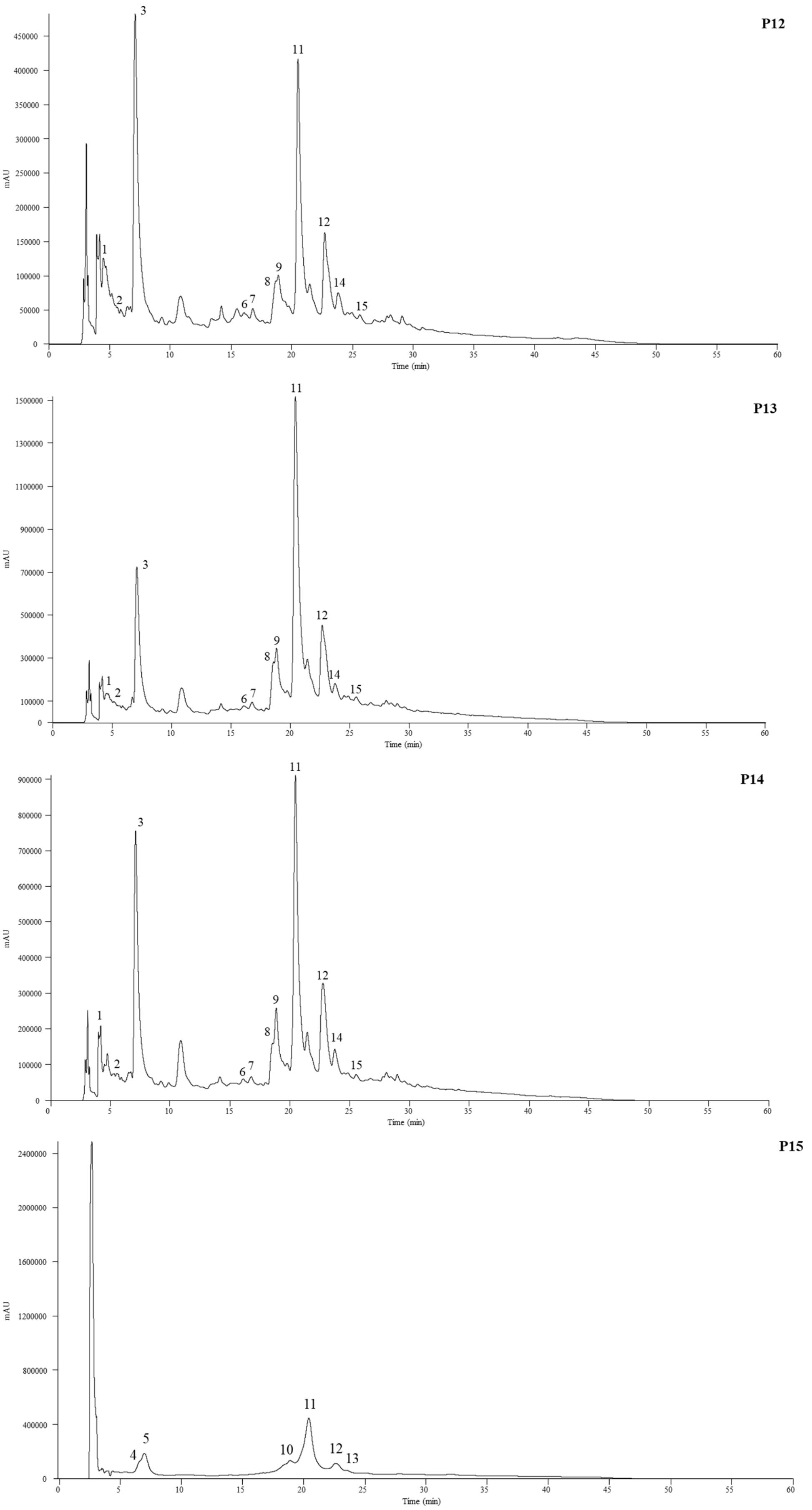
P12
mAU
Time (min)
1
2
3
6
7
8
9
11
12
14
15
P13
mAU
Time (min)
1
2
3
6
7
8
9
11
12
14
15
P14
mAU
Time (min)
1
2
3
6
7
8
9
11
12
14
15
P15
mAU
Time (min)
4
5
10
11
12
13

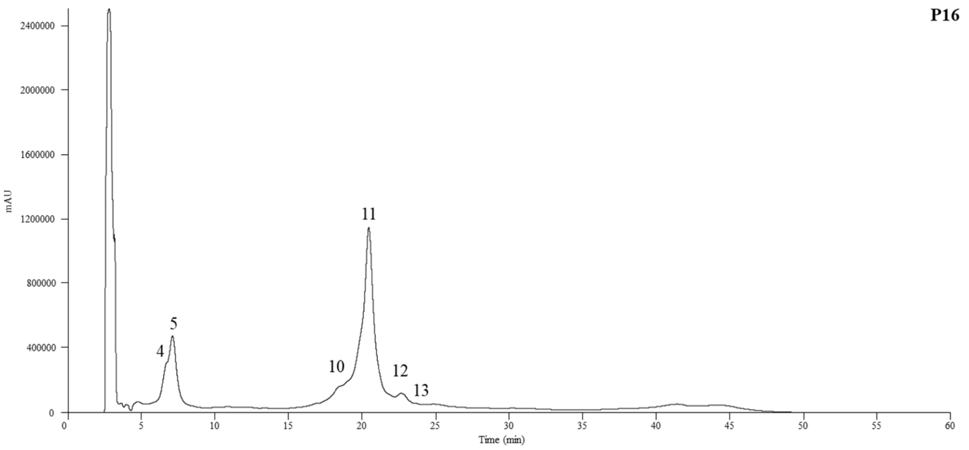

Supplementary Figure S1. Exemplificative phenolic profiles of the sixteen samples of cardoon studied, recorded at 280 nm.

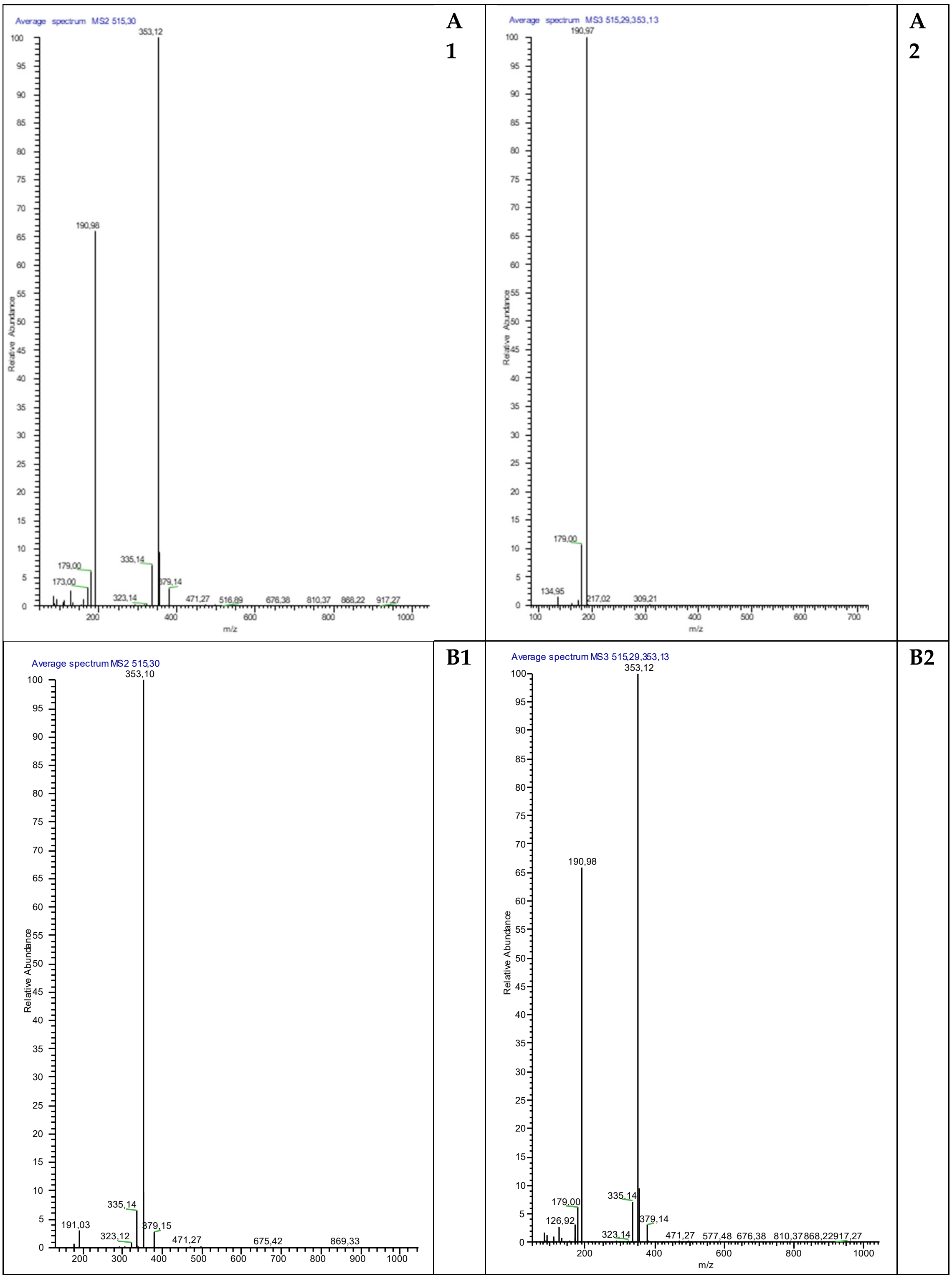
A
1
Average spectrum MS2 515,30
353,12
190,98
179,00
173,00
335,14
323,14
379,14
471,27
516,89
676,38
810,37
868,22
917,27
Relative Abundance
m/z
A
2
Average spectrum MS3 515,29,353,13
190,97
179,00
134,95
217,02
309,21
Relative Abundance
m/z
B1
Average spectrum MS2 515,30
353,10
335,14
191,03
323,12
379,15
471,27
675,42
869,33
Relative Abundance
B2
Average spectrum MS3 515,29,353,13
353,12
190,98
179,00
126,92
335,14
323,14
379,14
471,27
577,48
676,38
810,37
868,22
917,27
Relative Abundance
m/z

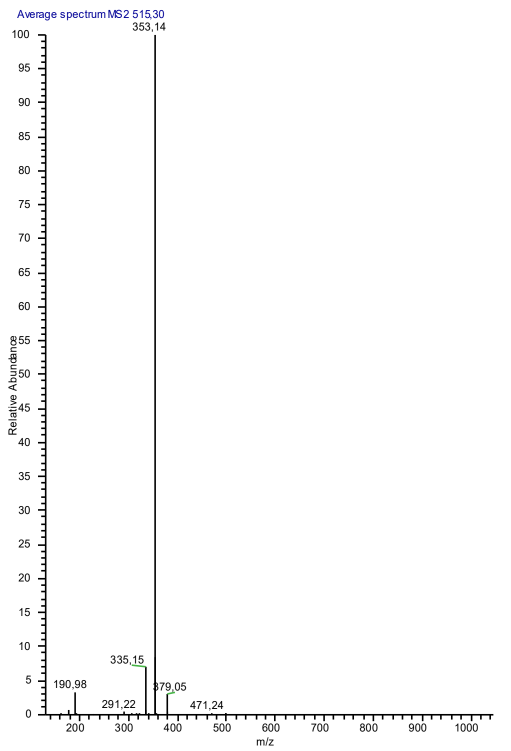
Average spectrum MS2 515,30
353,14
335,15
190,98
291,22
379,05
471,24
Relative Abundance
m/z

C1

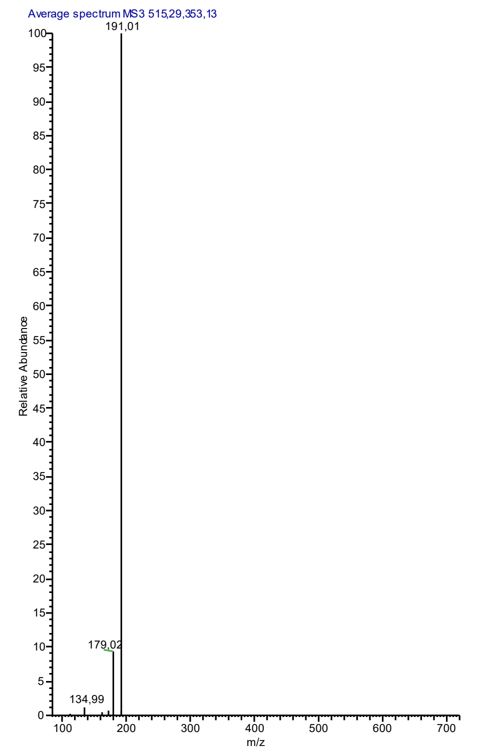
Average spectrum MS3 515,29,353,13
191,01
179,02
134,99
Relative Abundance
m/z

C2

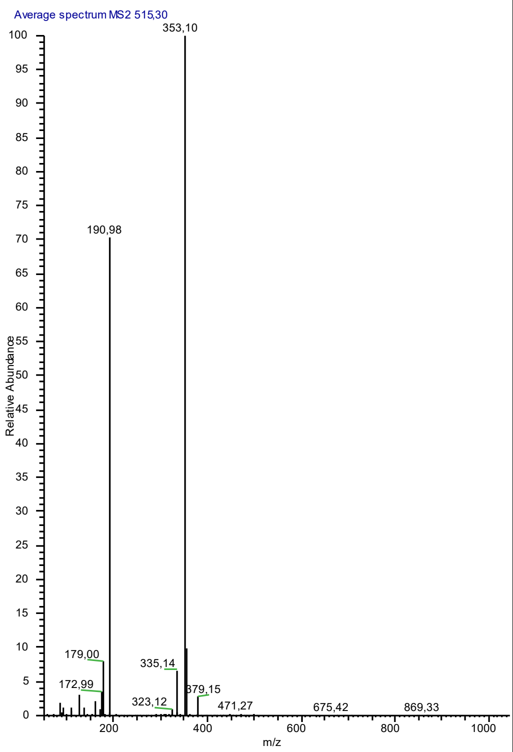
Average spectrum MS2 515,30
353,10
190,98
179,00
172,99
335,14
323,12
379,15
471,27
675,42
869,33
Relative Abundance
m/z

D1

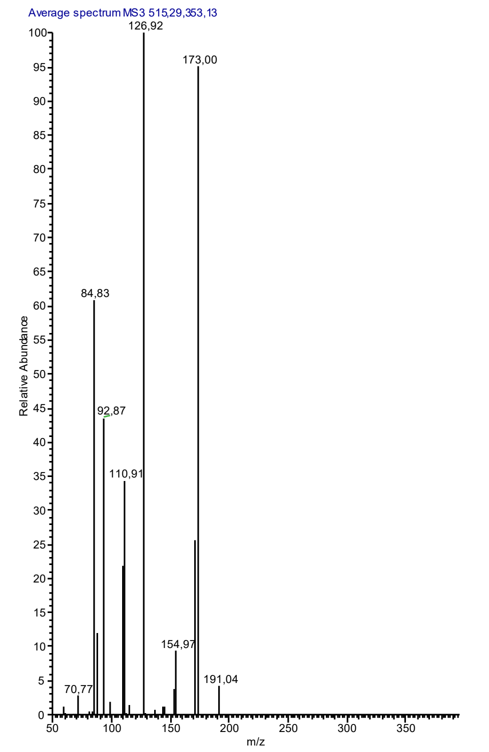
Average spectrum MS3 515,29,353,13
126,92
173,00
84,83
92,87
110,91
154,97
70,77
191,04
Relative Abundance
m/z

D2

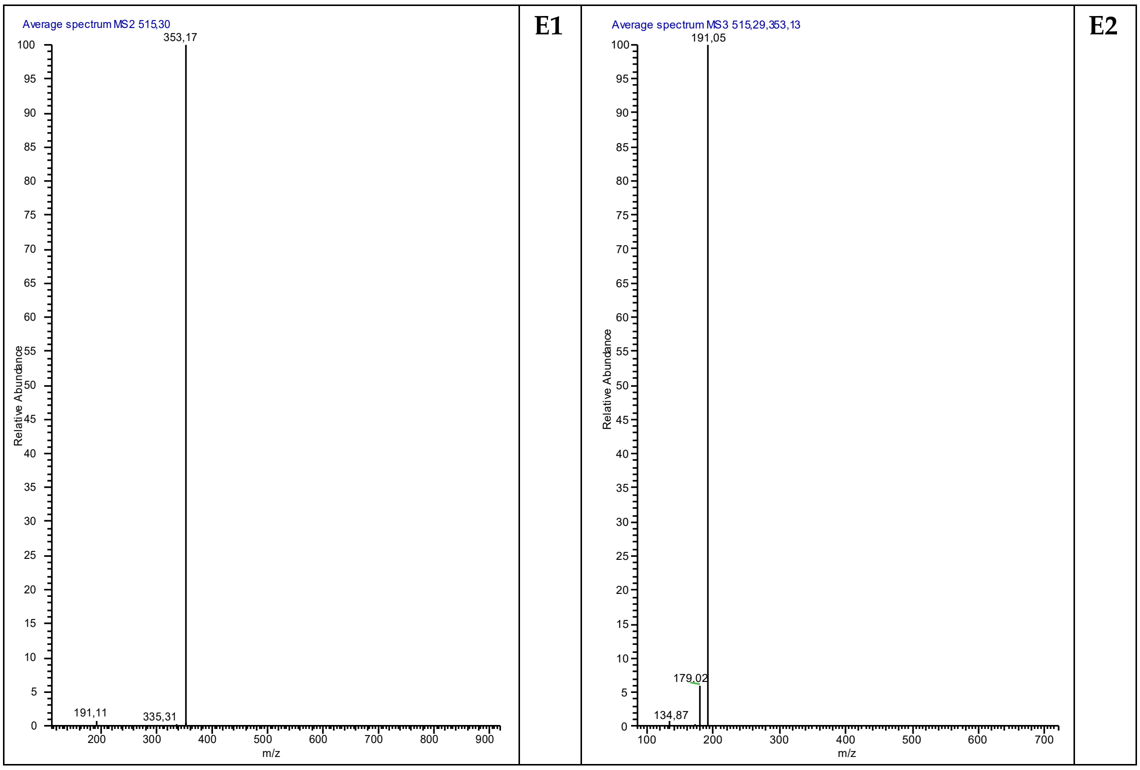

Supplementary Figure S2. MS^2 (**1**) and MS^3 (**2**) spectra of the dicaffeoylquinic acid isomers found in petioles samples of cardoon: **A1/A2** - 1,3-di-*O*-caffeoylquinic acid; **B1/B2** - *O*-dicaffeyolquinic acid; **C1/C2** - 1,5-di-*O*-cafffeoylquinic acid; **D1/D2** - 3,4-di-*O*-cafffeoylquinic acid; and **E1/E2** - 3,5-di-*O*-caffeolyquinic acid.
